# Fungus-mediated bacterial survival and migration enhance wood lignin degradation

**DOI:** 10.1128/aem.01347-25

**Published:** 2025-10-29

**Authors:** Ichiro Kamei, Kimiko Honsho

**Affiliations:** 1 Faculty of Agriculture, University of Miyazaki 12952, Miyazaki, Japan; Shanghai Jiao Tong University, Shanghai, China

**Keywords:** lignin degradation, bacterial-fungal interaction, co-existing bacteria, *Trametes versicolor*, white rot fungi

## Abstract

Bacterial–fungal interactions are increasingly recognized as a key factor in wood decomposition; however, the specific mechanisms underlying these interactions remain unclear. In this study, vanillic acid-utilizing bacteria were isolated from decayed wood colonized by the white-rot fungus *Trametes versicolor,* and their coexistence and functional impact in a solid wood environment were evaluated. We propose, for the first time, that these bacteria disperse on woody materials with fungal mycelia, survive long-term only in the presence of the fungus, and enhance both wood mass loss and lignin degradation in co-culture. Notably, the co-cultures exhibited lower glucose concentrations and vanillic acid levels released from the rotting wood powder than the fungal monocultures. These results suggest that bacterial glucose and vanillic acid consumption may relieve carbon catabolite repression, thereby promoting the fungal ligninolytic enzyme laccase. This study provides the first experimental evidence that coexisting bacteria can modulate fungal metabolism and wood-decay performance in solid-state systems via both physical and metabolic interactions.

**IMPORTANCE:**

White-rot fungi are central to lignin degradation in forest ecosystems; however, the ecological roles of coexisting bacteria in solid wood environments remain poorly understood. Here, we show that the white-rot fungus Trametes versicolor facilitates bacterial migration and long-term survival in wood, enabling bacteria to access fungal-derived aromatic and carbohydrate compounds. Additionally, these bacteria may enhance fungal ligninolytic activity by metabolizing phenolic and sugar intermediates that could otherwise accumulate and affect fungal metabolism. This mutualistic interaction suggests a spatially organized metabolic cooperation that may accelerate wood decomposition. Our findings highlight a novel perspective for bacterial-fungal interactions in structured lignocellulosic substrates and inform microbial strategies for efficient biomass degradation in natural and engineered systems.

## INTRODUCTION

Wood decay in forest ecosystems plays an important role in the global carbon cycle primarily through the microbial degradation of lignocellulosic biomass composed of cellulose, hemicellulose, and lignin. White-rot fungi are known for their ability to depolymerize lignin, a recalcitrant aromatic biopolymer in plant cell walls, using extracellular oxidative enzymes such as lignin peroxidases, manganese peroxidases, and laccases (1, 2). These enzymes produce various low-molecular-weight aromatic compounds as intermediates of lignin degradation, including vanillic acid (3, 4). Recent studies have demonstrated that such intermediates, particularly vanillic acid, can be further metabolized and utilized as carbon sources by white-rot fungi (5), suggesting that vanillic acid is a pivotal intermediate in lignin bioconversion processes.

Wood decay in natural environments is increasingly recognized as a collaborative process involving both fungi and bacteria. These microbial consortia form complex interactions that influence the lignocellulose degradation dynamics (6, 7). Previous studies have shown that fungi reduce the total number of cultivable bacteria in wood, suggesting a competitive relationship (8). However, recent research has revealed that some wood-associated bacteria exhibit nitrogen-fixing capabilities that can benefit ligninolytic fungi under nitrogen-poor conditions commonly found in woody substrates (9, 10). Although it has been suggested that bacteria may synergistically decompose lignin with fungi because of their potential to degrade lignin (11), and there have been reports of increased laccase production by white-rot fungi (12), no mechanism has been proposed to explain the effect of bacteria on promoting wood decay and lignin production by white-rot fungi. Despite these advances, the roles of bacterial partners in lignin decomposition remain poorly understood.

White-rot fungi are generally more efficient in degrading high-molecular-weight lignin polymers and oligomers, owing to their extracellular oxidative enzyme systems (1), whereas bacteria are often better adapted to catabolize low-molecular-weight lignin-derived aromatics such as vanillic acid and related compounds (13, 14). Therefore, the main role of bacterial catabolism in lignin degradation is estimated the mineralization of heterogeneous low-molecular-weight aromatics derived from lignin depolymerization by white-rot fungi (14). The catabolism of lignin-related aromatic compounds has been reported in several bacteria. In many cases, the pathway for the degradation of G-type lignin fragments by bacteria proceeds via vanillic acid. Although the bacterial catabolism of low-molecular-weight lignin-derived compounds is well-documented (14), most studies have been conducted in liquid media. The ability of bacteria to survive and function within the solid, recalcitrant matrix of wood remains largely unexplored. Furthermore, bacterial motility in wood, a nonaqueous and structurally complex environment, is severely limited, raising questions about how bacteria access new substrates or persist over time in such habitats.

Recent hypotheses suggest that bacteria in decaying wood may depend on fungal partners, not only for metabolic byproducts but also for physical scaffolding that facilitates colonization. This leads to the intriguing possibility that fungal mycelia provide both nutritional and structural support to bacteria, enabling them to survive and migrate within the wood matrix. This hypothesis is associated with the “fungal highway” model observed in soils (15).

In the present study, we isolated vanillic acid-utilizing bacteria from decaying wood colonized by *Trametes versicolor*, a common white-rot fungus often found in forest environments, and investigated their ecological relationship. We hypothesized that these bacteria rely on *T. versicolor* for both survival and dispersal within the wood environment and that such an interaction could enhance lignin degradation. Our objectives were to (i) assess bacterial survival in wood with and without fungal co-cultivation, (ii) evaluate bacterial migration along fungal hyphae, and (iii) determine the impact of bacterial–fungal interactions on lignin degradation efficiency.

## RESULTS

### Microbial community structure of decayed wood

**Fig 1:**
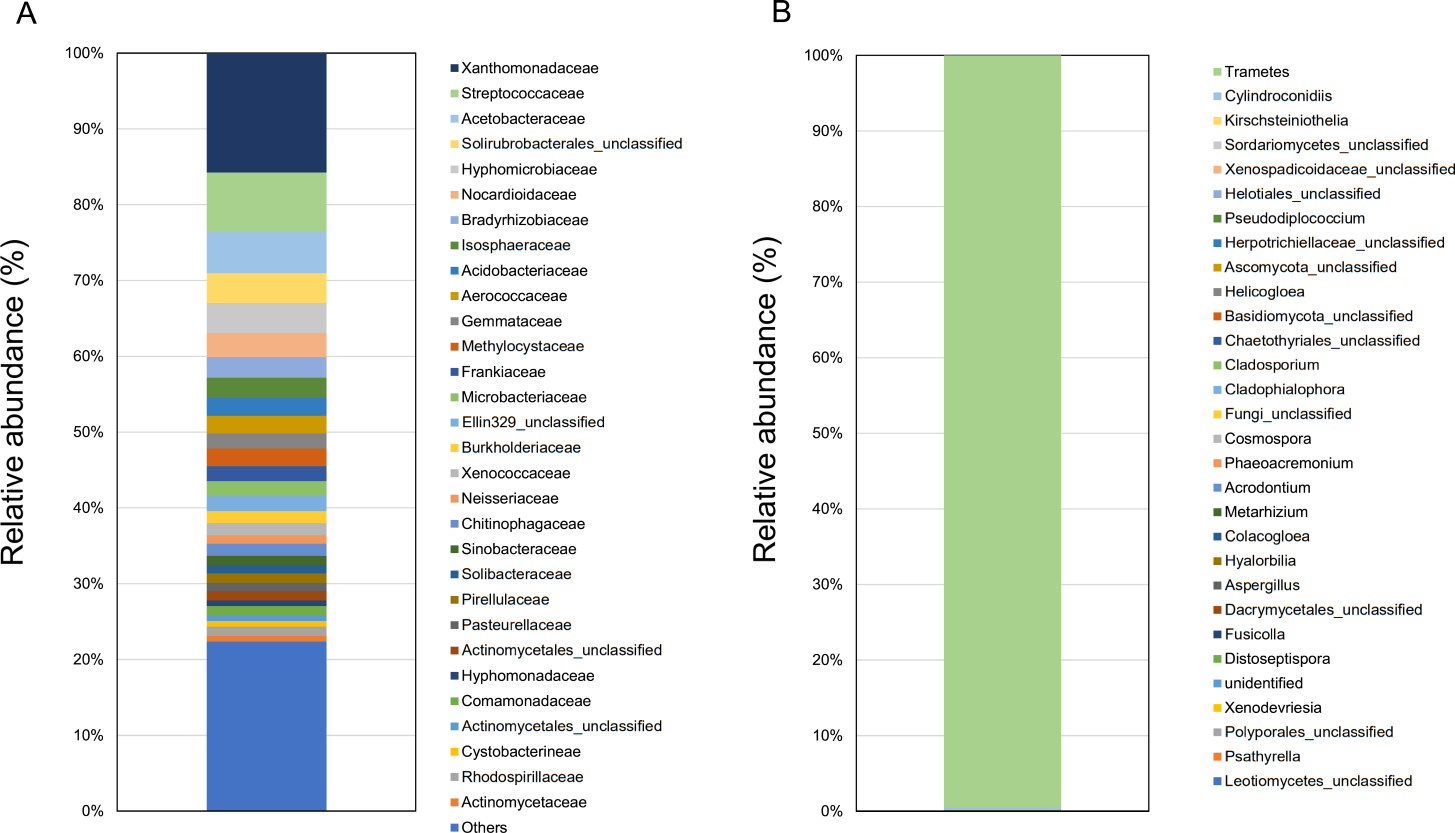
Bacterial and fungal community profiles of *Trametes versicolor* in rotting wood. (**A**) Prokaryotic community composition of rotting wood colonized by *T. versicolor* fruiting bodies at the family level. The top 30 families are shown. (**B**) Fungal community composition in rotting wood at the genus level.

High-throughput amplicon sequencing of the 16S rRNA gene (V3–V4 region) and the fungal ITS region revealed the microbial composition of the decaying wood sample. The bacterial communities were dominated by Proteobacteria (46.7%), Actinobacteria (19.0%), Firmicutes (11.0%), Planctomycetes (7.6%), and Acidobacteria (4.8%) (Fig. 1A). Among Proteobacteria, Xanthomonadaceae was the most abundant family. Fungal analysis showed that >98% of the reads belonged to the genus *Trametes*, indicating a strong dominance of *T. versicolor* (Fig. 1B).

### Isolation and selection of vanillic acid-utilizing bacteria

Seventeen bacterial strains capable of utilizing vanillic acid as the sole carbon source were isolated from the decayed wood sample (Table 1). These isolates grew on M9 agar with 5 mM vanillic acid and showed increased OD_600_ and vanillic acid consumption in liquid M9 medium after 3 days of incubation (Table 1). BLAST analysis of the 16S rRNA sequences identified them as belonging to the genera *Pandoraea*, *Burkholderia*, and *Pectobacterium*. Four strains (TN34, TN50, TN56, and TN59) were selected for further analyses. *In vitro* potato dextrose agar (PDA) confrontation assays revealed neither inhibition nor stimulation of *T. versicolor* mycelial growth by these bacterial strains (data not shown).

**TABLE 1:** Isolated and selected bacterial strains which utilize vanillic acid as carbon source from *T. versicolor* decayed wood

Strain no.	High identity with	Identity (%)	Vanillic acid degradation^*a*^
TN34^*b*^	*Pandoraea vervacti* strain NS15	99	50.0
TN35	*Pandoraea vervacti* strain NS15	99	47.3
TN36	*Pandoraea vervacti* strain NS15	99	52.1
TN37	*Pandoraea vervacti* strain NS15	99	59.0
TN38	*Pandoraea vervacti* strain NS15	99	40.6
TN39	*Pandoraea vervacti* strain NS15	99	60.1
TN40	*Pandoraea vervacti* strain NS15	99	56.7
TN46	*Pectobacterium cypripedii* strain B1	99	100
TN47	*Burkholderia nodosa* strain Br3461	98	61.4
TN48	*Burkholderia nodosa* strain Br3461	98	90.8
TN50^*b*^	*Burkholderia caballeronis* strain TNe-841	99	100
TN56^*b*^	*Burkholderia nodosa* strain Br3461	98	94.3
TN57	*Burkholderia nodosa* strain Br3470	98	30.5
TN58^*b*^	*Burkholderia nodosa* strain Br3470	98	46.8
TN59	*Pectobacterium cypripedii* strain B1	99	100
TN60	*Pandoraea vervacti* strain NS15	99	100
TN67	*Pandoraea vervacti* strain NS15	99	100

^*a*^ Vanillic acid decomposition rate in M9 medium containing 5 mM vanillic acid among 3 day incubation.

^*b*^ Bacterial strains selected for further experiments in the present study.

### Enhanced wood degradation in bacterial–fungal co-cultures

**Fig 2:**
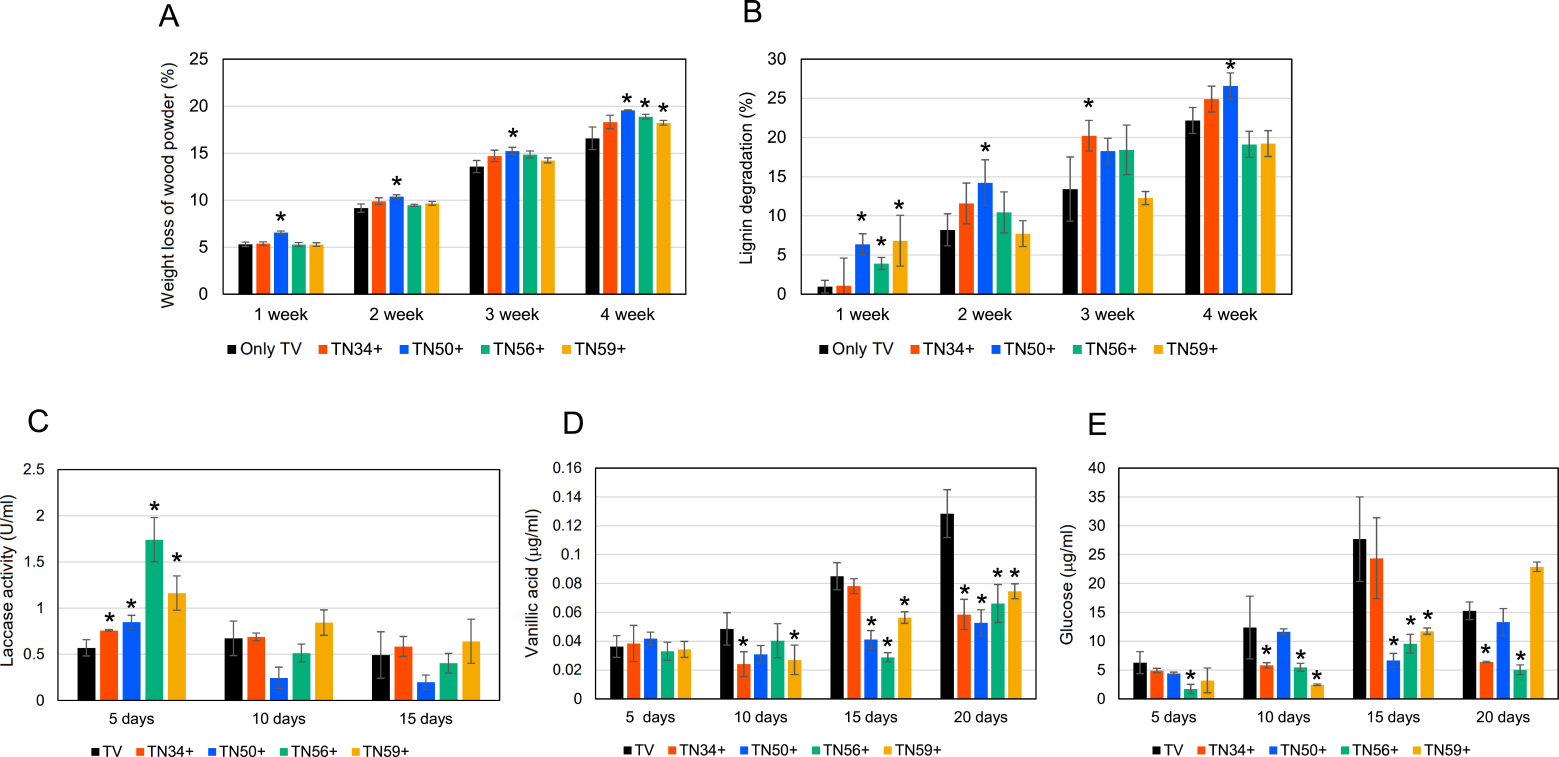
Effect of bacterial–fungal co-culture on wood powder decomposition. (**A**) Weight loss of wood powder after 4 week incubation. (**B**) Degree of lignin degradation calculated as Klason lignin loss. (**C**) Laccase activity measured from water extracts. Concentrations of vanillic acid (**D**) and glucose (**E**) in methanol extracts from wood powder medium. *T. versicolor* (TV) with bacterial co-cultures are denoted as TN34+, TN50+, TN56+, and TN59+. Data represent mean ± SD (*n* = 4 for A and B, *n* = 3 for C, D, and E). Asterisks indicate significant differences from fungal control TV (*P* < 0.05).

To assess the impact of bacterial co-culture on wood degradation by *T. versicolor*, the weight loss of wood powder (Fig. 2A) and lignin degradation (Fig. 2B) were measured over 4 weeks. Although *T. versicolor* alone caused approximately 16% wt loss, co-culture with TN50, TN56, or TN59 resulted in higher degradation across several time points. Notably, co-cultures with TN34 and TN50 exhibited significantly enhanced wood powder weight loss at 2 and 3 weeks compared with those of fungal monocultures.

Lignin degradation was not inhibited by the bacterial co-culture and was slightly enhanced in all combinations (Fig. 2B). A significant increase in lignin degradation was observed during the incubation (1 week) when TN50, TN56, or TN59 were co-cultured with *T. versicolor*. Higher laccase activity was detected on day 5 after co-cultivation of *T. versicolor* with bacterial strains TN50, TN56, or TN59 than when *T. versicolor* was cultured alone (Fig. 2C). No laccase activity was detected in wood meal cultures containing bacteria alone (data not shown), suggesting that the enhanced activity in co-culture was mainly of fungal origin.

*T. versicolor* cultures with TN34, TN50, TN56, and TN59 on R2A medium had viable bacterial cell counts of 1.2 × 10^6^, 4.0 × 10^5^, 1.9 × 10^6^, and 1.5 × 10^5^, respectively, after 2 weeks. No bacterial colonies were observed in fungal or bacterial monocultures at this time point.

Vanillic acid in the methanol extracts was below detectable levels in uninoculated wood powder and that after 5 days of incubation *T. versicolor*. However, detectable levels were observed after 10 days of fungal incubation, or 5 days of incubation after bacterial inoculation (Fig. 2D). Although vanillic acid accumulated in a time-dependent manner in the culture of *T. versicolor* alone, co-cultures with TN34, TN50, TN56, or TN59 resulted in a lower accumulation of vanillic acid across several time points, suggesting synergistic degradation of vanillic acid by coexisting bacteria (Fig. 2D). Glucose was observed in the degradation experiment using *T. versicolor*, indicating the hydrolysis of cellulose. During the bacterial and fungal co-incubation, the glucose concentration was maintained at a lower level than that of the *T. versicolor* monoculture (Fig. 2E).

### Prolonged bacterial survival in co-culture with *T. versicolor*

To evaluate bacterial survival in solid wood environments, colony-forming units (CFUs) were tracked over time in the co-cultures and monocultures (Table 2). In bacterial monocultures, viable cells were undetectable within 3–9 days, depending on the strain. In contrast, the co-cultures maintained detectable CFUs even after 30 days, indicating that the presence of fungi substantially prolonged bacterial survival in the wood. These findings indicated the severity of the wood environment for bacterial survival and the potential protective or supportive roles of *T. versicolor*. When the non-vanillic acid-utilizing bacteria *Escherichia coli* NBRC 3972 and *Enterobacter* sp. TN3W-14 were inoculated on wood medium, both strains failed to survive long-term in wood medium regardless of the presence or absence of *T. versicolor* (Table 2). *E. coli* NBRC 3972 tended to lose viability more rapidly in co-culture with *T. versicolor* than in the control without the fungus, suggesting a possible negative interaction.

**TABLE 2:** Effect of co-culture of bacterium and *T. versicolor* on bacterial survival in wood powder^*a*^

Bacterium	Fungus	CFU per conical tube					
		0 days	3 days	6 days	9 days	15 days	30 days
TN34		5.2 × 10^8^ ± 1.1 × 10^8^	0^*b*^	0	0	0	0
TN34	TV	6.1 × 10^8^ ± 1.8 × 10^8^	2.5 × 10^6^ ± 1.9 × 10^6^	7.5 × 10^4^ ± 5.4 × 10^4^	3.7 × 10^3^ ± 6.7 × 10^2^	3.8 × 10^5^ ± 6.3 × 10^4^	2.7 × 10^5^ ± 1.9 × 10^5^
TN50		2.9 × 10^8^ ± 7.8 × 10^7^	5.3 × 10^7^ ± 1.1 × 10^7^	0	0	0	0
TN50	TV	4.4 × 10^8^ ± 1.5 × 10^8^	4.7 × 10^4^ ± 1.8 × 10^4^	2.2 × 10^6^ ± 2.1 × 10^6^	–^*c*^	5.0 × 10^6^ ± 1.0 × 10^5^	1.2 × 10^6^ ± 4.1 × 10^5^
TN56		3.5 × 10^8^ ± 8.1 × 10^7^	1.0 × 10^7^ ± 1.1 × 10^6^	1.7 × 10^5^ ± 1.0 × 10^5^	0	0	0
TN56	TV	3.7 × 10^8^ ± 5.6 × 10^7^	1.4 × 10^5^ ± 4.6 × 10^4^	7.0 × 10^4^ ± 1.5 × 10^4^	4.1 × 10^6^ ± 2.9 × 10^6^	5.3 × 10^6^ ± 5.2 × 106^7^	6.4 × 10^4^ ± 3.5 × 10^4^
TN59		2.8 × 10^7^ ± 5.8 × 10^6^	0	0	0		0
TN59	TV	2.4 × 10^7^ ± 4.2 × 10^6^	1.5 × 10^3^ ± 5.0 × 10^2^	3.4 × 10^4^ ± 6.5 × 10^3^	2.0 × 10^6^ ± 5.1 × 10^5^		0
*E. coli*		1.3 × 10^8^ ± 3.0 × 10^7^	1.8 × 10^7^ ± 8.5 × 10^6^	–^*d*^	0	0	
*E. coli*	TV	1.4 × 10^8^ ± 4.0 × 10^8^	0	0	0	0	
TN3W-14		7.5 × 10^8^ ± 6.8 × 10^7^	–^*d*^	0	0	0	
TN3W-14	TV	9.3 × 10^8^ ± 1.1 × 10^8^	3.8 × 10^5^ ± 2.5 × 10^5^	0	0	0	

^*a*^ Bacterial survival was assessed after incubation in conical tube with wood powder medium. Following cultivation, each tube was extracted with 10 mL sterile water, and the suspension was spread on R2A agar plates to determine CFU. Values are expressed as CFU per a conical tube.

^*b*^ 0 indicates no colonies detected in any of 4 replicate tubes.

^*c*^ – indicates not quantifiable; colonies detected only in 2 of 4 replicate conical tubes (others yielded 0 CFU).

^*d*^ – indicates not quantifiable; colonies detected only in 1 of 4 replicate conical tubes (others yielded 0 CFU).

### Fungal mycelium facilitates bacterial dispersal in wood

**Fig 3:**
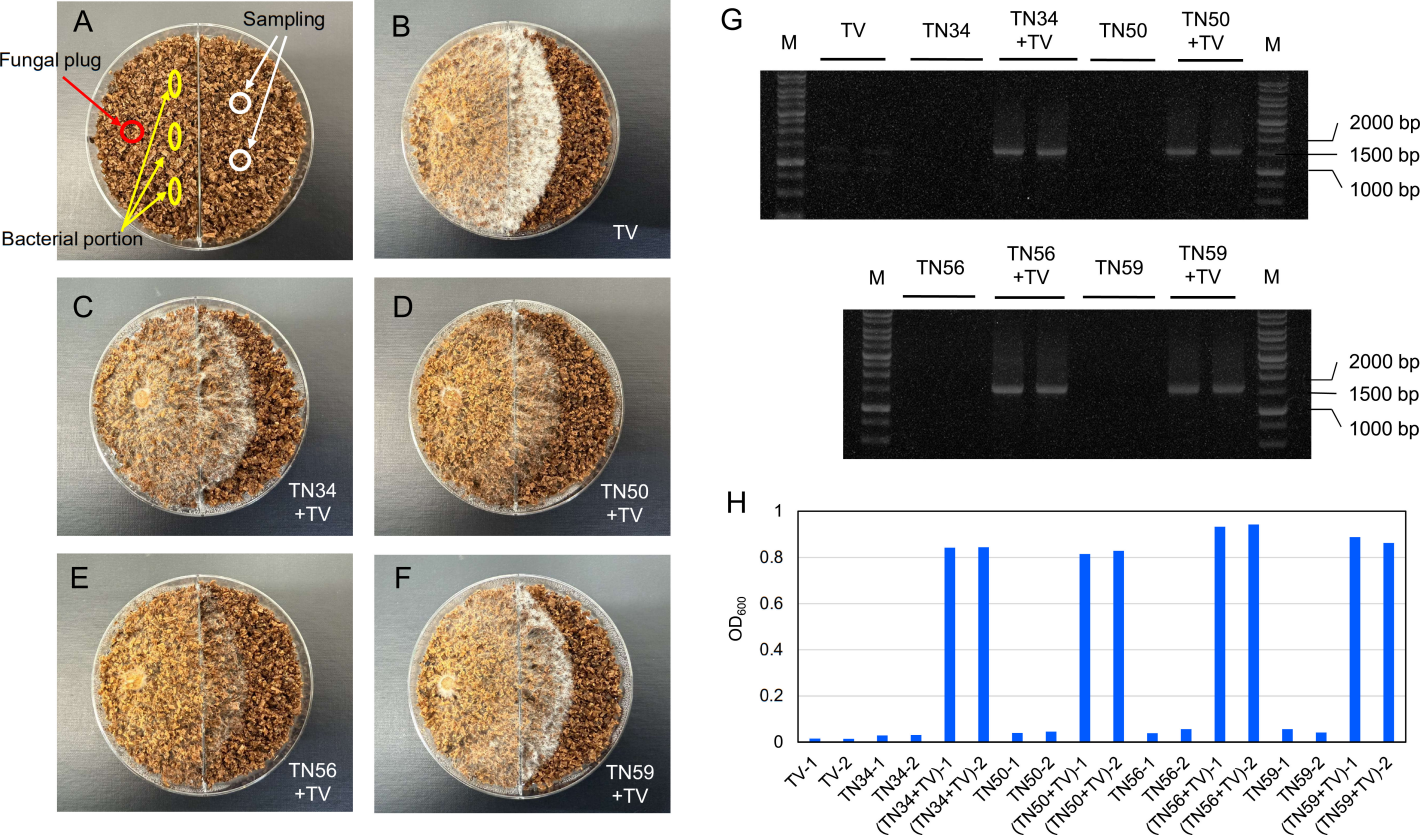
Evaluation of bacterial migration along fungal hyphae using a dual-chamber plate assay. (**A**) Inoculation sites of *T. versicolor* and bacteria on wood chip culture medium and the sampling sites for bacterial detection. (**B–F**) Mycelium growth at the time of sampling (7 days after bacterial inoculation) on wood chip medium. (**G**) PCR amplification of 16S rRNA genes from the opposite chamber confirmed bacterial presence. Two lanes represent two independent sampling sites from each coculture plate. TV: *T. versicolor* only; TN34, TN50, TN56, and TN59: bacteria only. (**H**) OD_600_ in the R2A liquid medium incubated for 3 d to confirm viable bacterial cells. −1 and −2 indicate the independent sampling sites.

A dual-chamber woodchip assay was used to assess bacterial movement along the fungal mycelia (Fig. 3A through F). *T. versicolor* mycelia crossed the partition into the adjacent chamber after 7 days. PCR amplification of bacterial 16S rRNA from hyphae in the second chamber confirmed the presence of bacteria only in the co-cultures (Fig. 3G). No signal was detected in monoculture controls of *T. versicolor* or in the bacterial strains. Additionally, samples from the hyphal tips showed visible growth in the R2A broth (Fig. 3H), further confirming bacterial viability and dispersal. In the case of non-vanillic acid-utilizing bacterial strains, *E. coli* NBRC 3972 showed no migration along *T. versicolor* mycelium. By contrast, *Enterobacter* sp. TN3W-14 was able to migrate along fungal hyphae to the opposite chamber (Fig. S1).

To assess long-term bacterial survival following fungal-assisted migration, we sampled the destination chambers of the dual-compartment plates at 30, 60, and 90 days after the start of co-culture. All tested strains exhibited growth in liquid R2A medium after 30 days, indicating successful survival (Fig. S2). At day 60, growth was observed for TN34, TN50, and TN56 (Fig. S3). At day 90, TN34 and partial TN56 showed detectable growth (Fig. S4). These observations suggest that some strains can not only migrate along fungal hyphae but also maintain viability in the wood substrate for extended periods following migration.

## DISCUSSION

In this study, we revealed a cooperative relationship between the white-rot fungus *T. versicolor* and vanillic acid–utilizing bacteria in wood environments. Our results demonstrate that co-cultivation of *T. versicolor* with selected bacterial strains not only enhances lignin and wood degradation but also enables long-term bacterial survival and dispersal in solid wood. These findings contribute to a better understanding of microbial interactions during wood decay and suggest a functional symbiosis that could be harnessed for efficient lignocellulose bioconversion.

### Bacterial community associated with *T. versicolor*

Among the Proteobacteria family, the most abundant was Xanthomonadaceae in wood rotted by *T. versicolor* in the present study. This dominant family is in accordance with previous reports on the bacterial community in decaying wood of esca-diseased grapevines (16), beech (17), and beech sawdust inoculated with *Phanerochaete chrysosporium* (18). Xanthomonadaceae have also been reported to degrade cellulose (19). Other dominant phyla, such as Actinobacteria, Firmicutes, and Planctomycetes, have been identified as potential members of the cellulose-degrading community in agricultural soil under oxic or anoxic conditions (20). Recent functional profiles of the wood log-inoculated white-rot fungus *Grifola frondosa* have indicated the existence of a higher relative abundance of cellulolysis, glycolysis, and the tricarboxylic acid cycle, indicating that bacterial communities may help cellulose degradation by *G. frondosa* in wood decay (21). These results, and those of previous reports, indicate that the bacterial community coexisting with white-rot fungi is strongly involved in carbohydrate degradation.

### Bacterial–fungal cooperation enhances wood and lignin degradation

White-rot fungi are known for their ability to degrade lignin via laccases and manganese peroxidases (1, 2). Although the lignin-degrading capacity of bacteria is relatively limited, some bacteria are known to catabolize low-molecular-weight aromatic intermediates, including vanillic acid (14, 22). In our study, the co-culture of *T. versicolor* with strains TN50, TN56, and TN59 significantly enhanced both wood weight loss and lignin degradation compared with fungal monocultures. This synergistic effect likely results from complementary metabolic activities, where fungi depolymerize lignin and bacteria metabolize aromatic byproducts, preventing feedback inhibition and enhancing the overall carbon flow. A few studies have shown that *in vitro* co-culture of bacteria and white-rot fungi promotes wood decay (11, 12).

One interesting finding of the present study is the marked reduction in glucose concentration in the wood powder medium observed in the co-culture of *T. versicolor* and bacteria compared to that in the fungal monoculture. Since glucose is a product of cellulose hydrolysis by white-rot fungi, a decrease in glucose suggests that the bacteria assimilate the glucose released by the fungus. Glucose depletion affects fungal metabolism, including lignin degradation. The production of ligninolytic enzymes in white-rot fungi is subject to carbon catabolite repression (CCR), whereby the presence of sugars such as glucose suppresses the transcription of ligninolytic enzyme genes (23). Therefore, one possible mechanism for the enhancement of wood decay by bacteria observed in this study is that the coexisting bacteria indirectly released the fungus from the CCR by reducing glucose levels continuously. Bacteria may facilitate fungal transition from primary to secondary metabolism (lignin degradation).

Vanillic acid levels were lower in the co-culture treatments than in *T. versicolor* monoculture (Fig. 2D), suggesting the bacterial uptake of vanillic acid derived from lignin degradation by *T. versicolor*. Although certain phenolic intermediates have been reported to stimulate ligninolytic enzyme expression in white-rot fungi (24), their accumulation may alter the redox environment or impose a metabolic burden when present in excess. Recent evidence suggests that lignin-derived phenolic compounds, including vanillic acid, can act as competitive inhibitors of ligninolytic enzymes, such as laccases, thereby reducing degradation efficiency (25, 26). Moreover, these compounds have been shown to interfere with the cellulolytic processes, necessitating their enzymatic removal for effective biomass conversion (27, 28). Thus, the removal of vanillic acid by bacteria may help maintain a more favorable microenvironment for continued fungal activity, representing potential metabolic cooperation rather than a direct relief of repression.

Although the observed reduction in vanillic acid concentration in the co-culture treatments was not drastic, it provided significant ecological insight. In the present solid-state wood powder culture, vanillic acid is unlikely to be homogeneously distributed because it is generated locally during fungal lignin decomposition. Therefore, the bacterial uptake of such intermediates likely depends on their physical proximity and motility, rather than their diffusion through the liquid phase. This highlights the importance of bacterial migration along fungal hyphae not only for colonization but also for accessing spatially constrained metabolic niches.

This concept is consistent with findings from soil bioremediation studies, in which bacterial colonization and motility in structured habitats are critical for the degradation of polyaromatic hydrocarbons and other hydrophobic pollutants (29, 30). Fungal hyphae have been shown to act as conduits for bacterial transport, enabling them to overcome the limitations of spatial heterogeneity and low pollutant solubility. Similarly, our study suggests that, in lignocellulosic environments, bacterial movement along fungal networks facilitates access to dispersed aromatic compounds, thus forming a functional microbial consortium under solid-phase conditions.

### Fungal presence is essential for bacterial persistence in solid wood

Wood is nutrient-limited, with a high C/N ratio, rich in hydrophobic regions due to lignin and extractive components, and a compact structure in the cell wall, which is generally unfavorable for bacterial survival. Our results showed that bacteria could not survive for longer than a few days in either extracted wood powder or unextracted wood chip cultures when inoculated alone, indicating a very severe environment for the survival of bacteria. However, in the presence of *T. versicolor*, bacterial strains TN34, TN50, and TN56 persisted for over 30 and 60 days, indicating that the fungus supports bacterial survival. Compared to the other strains, TN59 had a shorter viability period when co-cultured with *T. versicolor*. The same trend was observed in both the extracted wood powder and unextracted wood chip media, indicating that the extractive components of wood had little effect on bacterial survival. In the wood powder degradation results, the enhancement of lignin degradation by TN59 was lower in the later stages of incubation, which is consistent with the lower survival of TN59. These results strongly suggest that wood decay of white-rot fungi by coexisting bacteria is strongly linked to bacterial survival and supports the hypothesis mentioned above that the role of bacteria as a “microbial sink” for glucose and vanillic acid promotes lignin degradation (31).

This phenomenon can be explained using several mechanisms. First, nutrients such as glucose and vanillic acid are supplied through fungal wood degradation. Second, the alteration of environmental conditions, such as moisture retention, pH, and loss of dense cell wall structure due to fungal activity, may be possible. Potential shelters within the hyphal structures have also been proposed. These findings expand on earlier work on soil fungi–bacteria interactions and provide clear evidence for fungal facilitation of bacterial survival in a highly recalcitrant wood matrix (32).

### Bacterial migration via fungal hyphae in wood environment

A novel finding of this study was the demonstration of bacterial movement with fungal hyphae through solid wood. While the “fungal highway” concept has been established in soil microbiology (15, 33), its occurrence in lignocellulose-rich environments such as wood has remained speculative. In aqueous conditions, bacterial migration would not rely on hyphal transport, and passive diffusion or active swimming might dominate. Our study focuses on solid woody substrates, where fungal-mediated migration is more critical. Our dual-chamber assay confirmed that bacterial DNA and viable cells appeared in the wood compartments only after hyphal invasion by *T. versicolor*, providing direct evidence that fungal mycelia act as dispersal pathways. In our study, bacterial motility was frequently observed on wood powder media but was highly limited or unstable on agar media (PDA or only agar medium). This suggests that the surface hydrophobicity plays a crucial role in facilitating bacterial dispersal in the presence of fungal mycelia. Fungal hyphae are known to exhibit hydrophobic characteristics (34) that can reduce water film formation and potentially create surface conditions favorable for fungal-guided bacterial migration. Similar findings have been reported for soil systems formed on hydrophobic surfaces, which enhanced the dispersal of bacteria through otherwise water-unsaturated environments. Kohlmeier (15) demonstrated that bacterial movement along fungal hyphae is promoted in conditions where water is limited, and the fungus bridges the gaps between hydrated microenvironments. These results indicate that the physical surface properties of fungal hyphae are critical for bacterial motility under non-aqueous conditions. In the context of our wood powder system, the combination of fungal hyphae and the hydrophobic nature of the lignocellulosic substrate may have created a conducive environment for bacterial dispersal. In contrast, agar, which is highly hydrophilic, may disrupt such interactions owing to excessive moisture and a lack of surface tension, which is conducive to surface-bound bacterial movement. This finding has ecological implications: it suggests that fungi not only chemically modify their environment but also physically structure microbial communities in wood by acting as vectors for bacterial migration. Such a strategy may enable spatially separated microbial partners to cooperate in degrading heterogeneous substrates, such as wood.

Despite providing clear evidence of fungal-mediated bacterial migration and survival, this study has inherent limitations. The physical association between bacterial cells and fungal hyphae could not be directly visualized, likely due to the low abundance of bacteria and the disruption of delicate interactions during conventional fixation and staining procedures. Future work incorporating fluorescently tagged bacteria will be essential to trace real-time spatial dynamics *in situ*. Moreover, while we focused on single bacterial strains under defined conditions, natural lignocellulosic environments involve far more diverse microbial communities. Expanding this research to multispecies systems will be key to unraveling the complexity and ecological significance of fungal–bacterial interactions in nature.

### Bacterial specificity for the migration and survival via fungal hyphae in wood environment

The additional experiments with non-vanillic acid-utilizing bacteria further clarify the specificity of the observed interactions. *E. coli* NBRC 3972 neither migrated along fungal hyphae nor survived on wood chip medium, supporting the notion that such traits are not universal among bacteria. Interestingly, *Enterobacter* sp. TN3W-14, which was previously reported to enhance the growth of white rot fungus *Phlebia brevispora* (35), was capable of migrating along *T. versicolor* hyphae, yet it failed to persist in wood chip medium even in the presence of fungal hyphae. This suggests that while hyphal migration may be a more general phenomenon among certain bacterial groups as proposed in the “fungal highway” concept, long-term survival on lignocellulosic substrates may require metabolic compatibility with the fungal host, particularly the ability to utilize fungal-derived aromatic compounds such as vanillic acid. These findings strengthen our conclusion that the co-survival observed in this study is specific to some bacteria and may be linked to their metabolic roles during wood decomposition.

### Conclusion

This study provided the first experimental demonstration that vanillic acid-utilizing bacteria can disperse within *T. versicolor* mycelia, persist in solid wood for extended periods, and synergistically enhance wood decomposition and lignin degradation. Our findings suggest that fungal mycelia serve not only as a structural source of movement but also as ecological scaffolds that support bacterial survival and function. Furthermore, we propose for the first time that bacterial consumption of glucose and lignin degradation products may alleviate CCR in white-rot fungi, thereby facilitating the induction of lignin-degrading enzymes. This dual role of bacteria as metabolic partners and physical co-migrants reveals a novel dimension of bacterial–fungal cooperation in wood ecosystems, with potential implications for biotechnological applications in biorefinery and biomass valorization.

## MATERIALS AND METHODS

### Sample collection and isolation of fungus and bacteria

The fruiting bodies of *T. versicolor* and the associated decayed hardwood were collected from the Tano Forest Science Station, University of Miyazaki, Japan. The decayed xylem (to a depth of approximately 2 cm) of the fallen *Castanopsis sieboldii* was excised after removing the surface bark. To isolate culturable bacteria, the wood chips were suspended in sterile water, vortexed, and plated in serial dilutions on M9 agar supplemented with 5 mM vanillic acid as the sole carbon source. Plates were incubated at 28°C for 2–7 days, and colonies were subsequently isolated. Bacterial utilization of vanillic acid was confirmed in liquid M9 medium containing 5 mM vanillic acid by monitoring OD_600_ and the depletion of vanillic acid over 3 days using high-performance liquid chromatography (HPLC). The basidiomycetous mycelium of *T. versicolor* was isolated from the fruiting body as previously described (11).

### Microbial community analysis

Total DNA was extracted from decayed wood using ISOFECAL (Nippon Gene Co., Ltd., Japan) following bead-beating and purified with LabAid824s (Zeesan Biotech Co., Ltd., China). For bacterial 16S rRNA gene amplification, a two-step tailed PCR was performed using the universal primers 341f and 805r. The primers ITS1-F_KYO1 and ITS2_KYO2 were used to amplify the fungal ITS region. PCR amplicons were sequenced on the Illumina MiSeq platform (Illumina, San Diego, CA, USA) with 2 × 300 bp paired-end reads using the MiSeq Reagent Kit v3 (Bioengineering Lab Co., Ltd., Japan). Sequence processing was performed using the FASTX-Toolkit (v0.0.14) and dada2 with operational taxonomical units clustered at 97% identity using QIIME2 (v2022.8).

### Co-cultivation of fungus and bacteria on wood powder

The experimental design is shown in Fig. S4. Extracts-free *Quercus serrata* wood powder (45–100 mesh, ~10% moisture content, 17.2% lignin contents) was adjusted to 75% moisture content by adding distilled water and autoclaving. *T. versicolor* was inoculated via a 9 mm mycelial plug and incubated at 28°C for 5 days. The PDA plug was then removed, and 100 µL of bacterial suspension (OD_600_ ≈ 1.0) was prepared by washing with sterile water twice from a 24-h R2A broth culture incubated at 28°C was inoculated. The control flasks were placed in sterile water. Flasks were sealed with a silicone stopper and incubated at 28°C for 30 days. After incubation, samples were dried at 105°C, and weight loss was calculated. Lignin content was determined according to a previous study (11) via acid hydrolysis using 72% sulfuric acid diluted to 4%, autoclaved at 121°C for 1 h, and analyzed gravimetrically from the insoluble fraction as Klason lignin. Lignin degradation (%) was calculated by comparing the lignin content of each sample after fungal or co-culture incubation with the initial lignin content of the untreated wood meal (based on dry weight). Values are shown as percentage reduction at each time point. Wood powder degradation assays were conducted in four separate Erlenmeyer flasks for each condition, and statistical analyses were performed using Dunnett’s test to compare each bacterial co-culture with the control (*T. versicolor* alone).

### Enzyme activity assays and vanillic acid and glucose determination

Incubated wood powder medium (prepared in the same manner as above) was extracted with 10 mL sterile water (4°C, 10 min). Supernatants were collected by centrifugation (14,000 × *g*, 4°C, 20 min). Laccase activity was measured based on the oxidation of 2,6-dimethoxyphenol at 469 nm. Portions of the extracts were plated onto R2A agar to confirm bacterial viability. The incubated wood powder (as described above) was freeze-dried and extracted with 10 mL of methanol to extract low-molecular-weight compounds. After centrifugation (14,000 × *g*, 20 min), the supernatant was collected and dried under a N_2_ stream. The extracts were analyzed by gas chromatography-mass spectrometry (GC/MS) after trimethylsilyl (TMS) derivatization. After the addition of N,O-bis(trimethylsilyl)trifluoroacetamide containing 1% trimethylchlorosilane and pyridine, the sample was reacted at 150°C for 10 min and then injected into the GC/MS system. All experiments using wood powder cultures were performed in three independent Erlenmeyer flasks per treatment. Statistical analyses were performed using Dunnett’s test to compare each bacterial co-culture with the control (*T. versicolor* alone).

### Evaluation of bacterial survival on wood chips

The experimental design is shown in Fig. S5. Unextracted *Quercus acutissima* wood chips (approximately 5 mm) were adjusted to 70% moisture by the addition of distilled water. Then, 4 g of wood chip (wet weight) was packed into 15 mL conical tubes sealed with silico stoppers and autoclaved. *T. versicolor* mycelial plugs were inoculated and incubated for 5 days. After plug removal, 50 µL of bacterial cell suspension in sterile water prepared as described above was added. Cultures were incubated at 28°C for up to 30 days. Samples were collected on days 0, 3, 6, 9, 15, and 30, suspended in 10 mL of sterile water, and plated onto R2A agar for colony counting. The control tubes did not contain any fungal inoculum. Survival assays were conducted in triplicate tubes per condition, and CFU data are shown as mean ± standard deviation. Statistical significance tests were not applied since the focus was on detecting viable bacterial cells.

### Bacterial migration assay on wood chips

Unextracted oak wood chips (~70% moisture, prepared in the same manner as mentioned above) were autoclaved and placed in two-chamber petri dishes. *T. versicolor* was inoculated into one chamber and incubated for 5 days until the hyphae approached the partition. Then, 10 µL of bacterial suspension was added at the hyphal front, and incubation continued for 7 days. Mycelia with wood chips from two sampling sites in the opposite chamber were collected, and DNA was extracted using the DNeasy PowerSoil Pro Kit (QIAGEN). Bacterial presence was confirmed by PCR of the 16S rRNA gene using primers 27f and 1525r (36) and by culturing in R2A broth with OD_600_ monitoring. The wood chip medium incubation was extended up to days 30 and 60, the mycelia with wood chips were sampled and inoculated into the R2A broth medium, and bacterial growth was monitored at OD_600_. From the culture that confirmed the growth of bacteria, DNA was extracted, and 16S rRNA gene sequencing was carried out to confirm the growth of the initial inoculated bacterial strain. Figure images were from single plates. Similar migration behavior was consistently reproduced in separate replicate experiments, confirming the reproducibility of the observations.

### Co-culture assays with non-vanillic acid-degrading bacteria

To test whether migration and long-term survival were specific to vanillic acid-utilizing bacteria, two additional bacterial strains were examined: *Escherichia coli* strain NBRC 3972 and *Enterobacter* sp. strain TN3W-14, which was originally isolated from decayed wood colonized by *Phlebia brevispora* and previously shown to promote fungal growth (35). Each strain was co-cultured with *T. versicolor* on wood chip medium using the same procedures described above for survival and migration assays.

### Analytical methods

HPLC was used to quantify vanillic acid in the liquid culture and to evaluate bacterial consumption. HPLC was performed using a Shimadzu LC-20AD fitted with an ODS-4 column with an inner diameter of 250 × 4.6 mm (GL Science Inc., Tokyo, Japan) and an SPD-20A UV/VIS detector. Water/methanol (1:1) was used as the solvent at a flow rate of 1.0 mL min^−1^. The compounds in the eluate were detected at 254 nm. GC/MS was used to analyze vanillic acid and glucose in the wood powder medium. GC/MS was performed using an Agilent Technologies 5975C system linked to an Agilent Technologies 7890A system equipped with an Agilent Technologies HP-5MS column (60 m  ×  0.250 mm). The oven temperature was programmed to increase from 80 to 300°C at 20°C min^−1^. Vanillic acid was determined using the selected ion monitoring mode at *m*/*z* 312. Glucose was detected in the scan mode. Since α- and β-anomers of glucose were observed as separate peaks due to TMS derivatization, their combined peak areas were used for quantification.

## Data Availability

The raw sequence data from bacterial and fungal community analyses have been deposited in the DDBJ Sequence Read Archive under BioProject accession numbers PRJDB36332 (bacterial 16S rRNA amplicons; Run accession DRR727329) and PRJDB37394 (fungal ITS amplicons; Run accession DRR727330). The 16S rRNA gene sequences of the bacterial isolates obtained in this study have been deposited in DDBJ under accession numbers LC888449–LC888465.

## SUPPLEMENTAL MATERIAL

Supplemental figures aem.01347-25-s0001.docx Figures S1 to S6.

ASM does not own the copyrights to Supplemental Material that may be linked to, or accessed through, an article. The authors have granted ASM a non-exclusive, world-wide license to publish the Supplemental Material files. Please contact the corresponding author directly for reuse.
