# Supplementary material for: Fungus-mediated bacterial survival and migration enhance wood lignin degradation
Source: Appl Environ Microbiol. 2025 Oct 29;91(11):e01347-25. doi: 10.1128/aem.01347-25 (PMC12628761; doi:10.1128/aem.01347-25)
Supplement: Supplemental figures — Figures S1 to S6. [file aem.01347-25-s0001.docx]

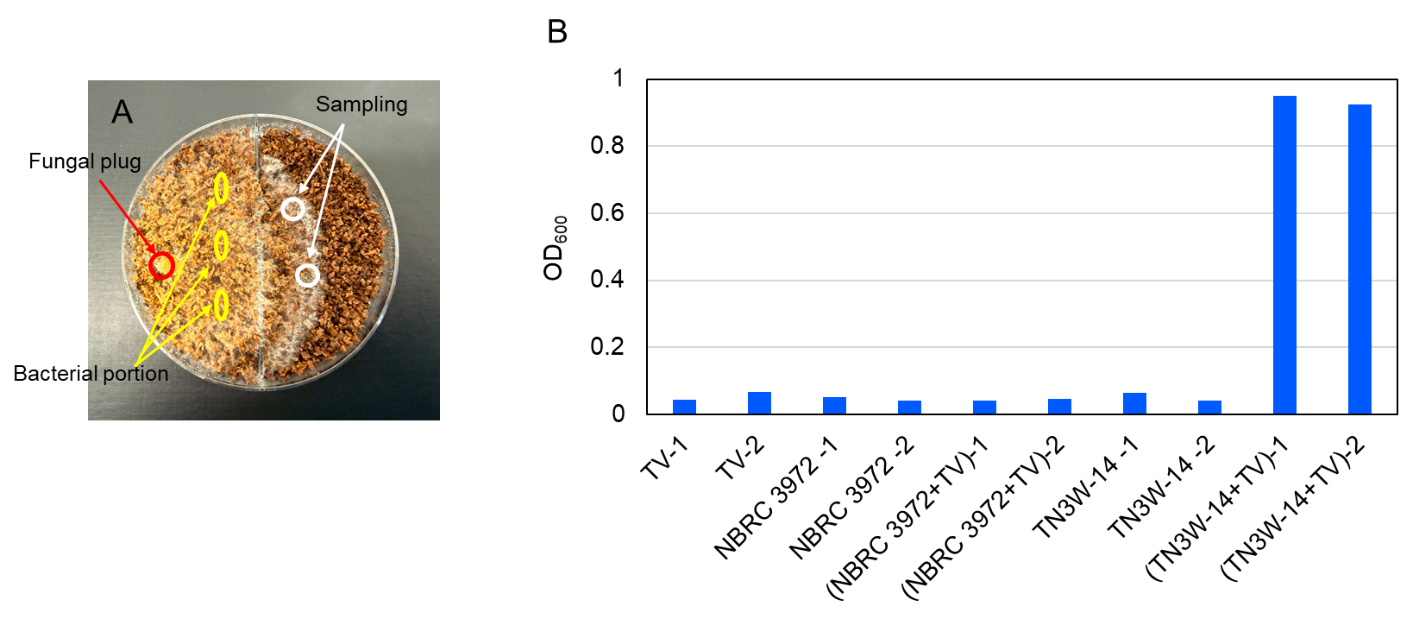


**Fig. S1**

Non-vanillic acid-utilizing bacterial migration along fungal hyphae using a dual-chamber plate assay. *E. coli* strain NBRC 3972 and *Enterobacter* sp. strain TN3W-14 were used. (A) inoculation sites of *T. versicolor* and non-vanillic acid-utilizing bacteria on wood chip culture medium and the sampling sites for bacterial detection. (B) OD_600_ in the R2A liquid medium incubated for 3 d to confirm viable bacterial cells. -1 and -2 indicate the independent sampling sites.


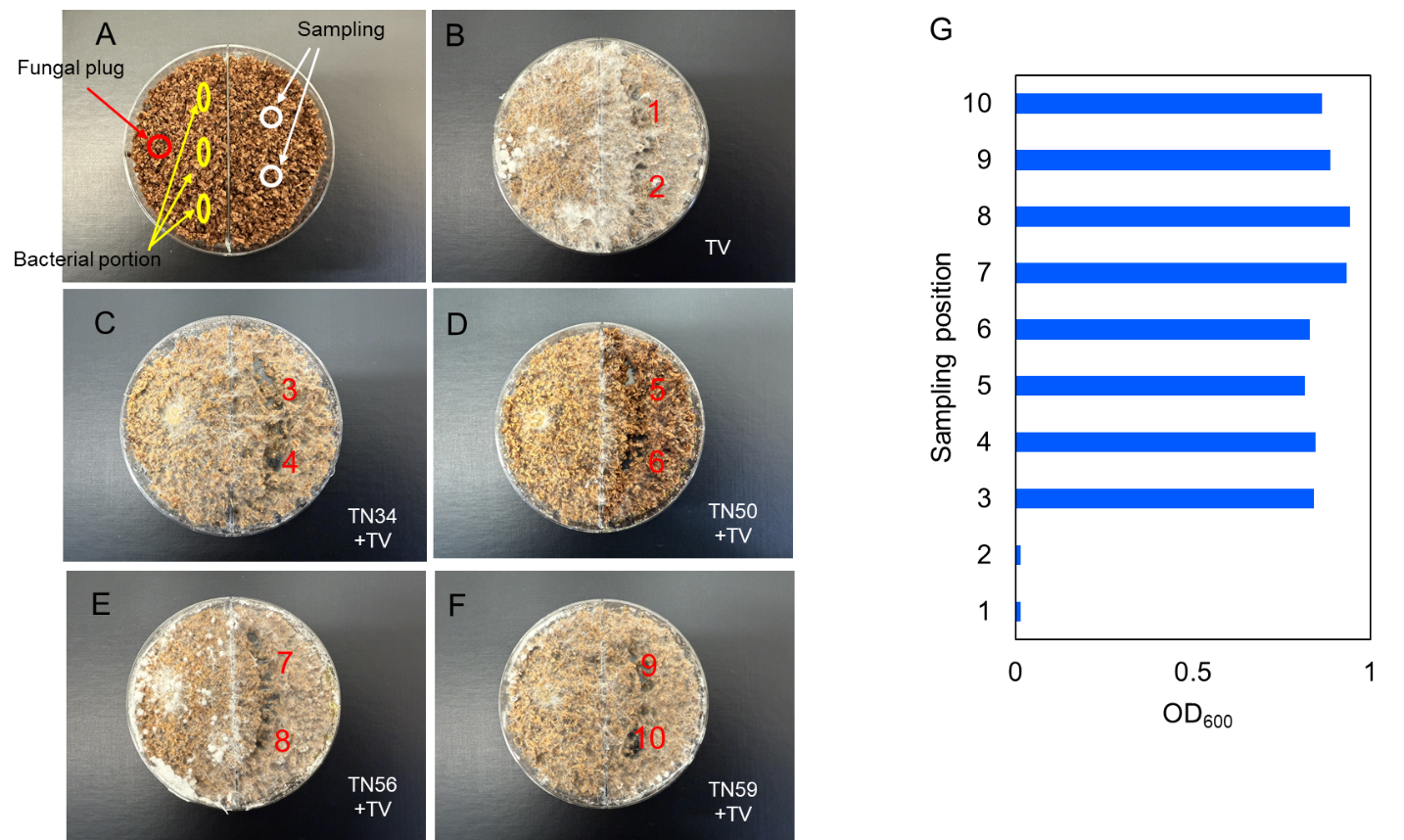


**Fig. S2**

Bacterial migration and survival along fungal hyphae using a dual-chamber plate assay for 30 d. (B-F) Mycelium growth at the time of sampling (30 d after bacterial inoculation) on wood chip medium. (G) OD_600_ in the R2A liquid medium incubated for 3 d to confirm viable bacterial cells of each sampling site. The number indicate the sampling position showing at B-F.


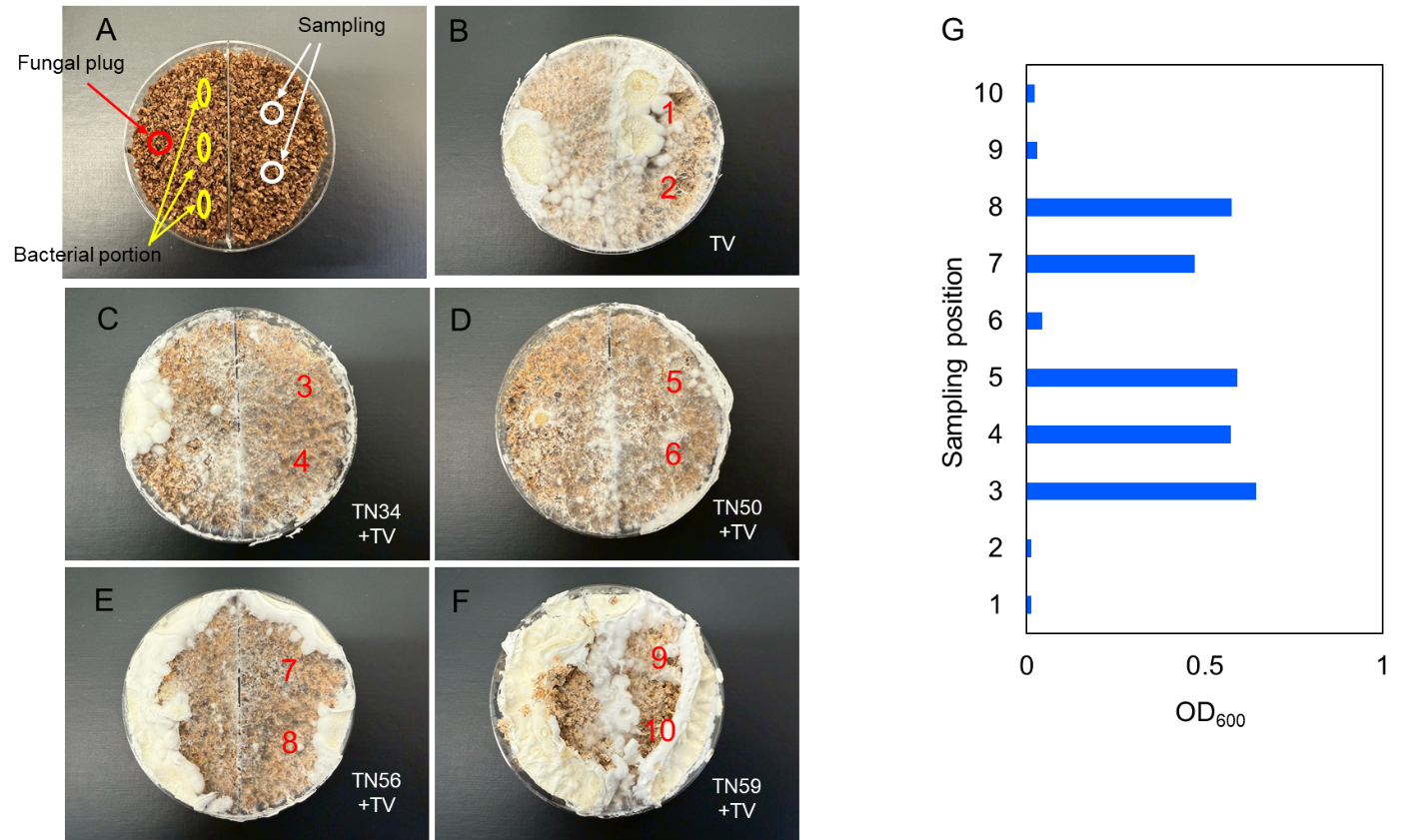


**Fig. S3**

Bacterial migration and survival along fungal hyphae using a dual-chamber plate assay for 60 d. (B-F) Mycelium growth at the time of sampling (60 d after bacterial inoculation) on wood chip medium. (G) OD_600_ in the R2A liquid medium incubated for 3 d to confirm viable bacterial cells of each sampling site. The number indicate the sampling position showing at B-F.


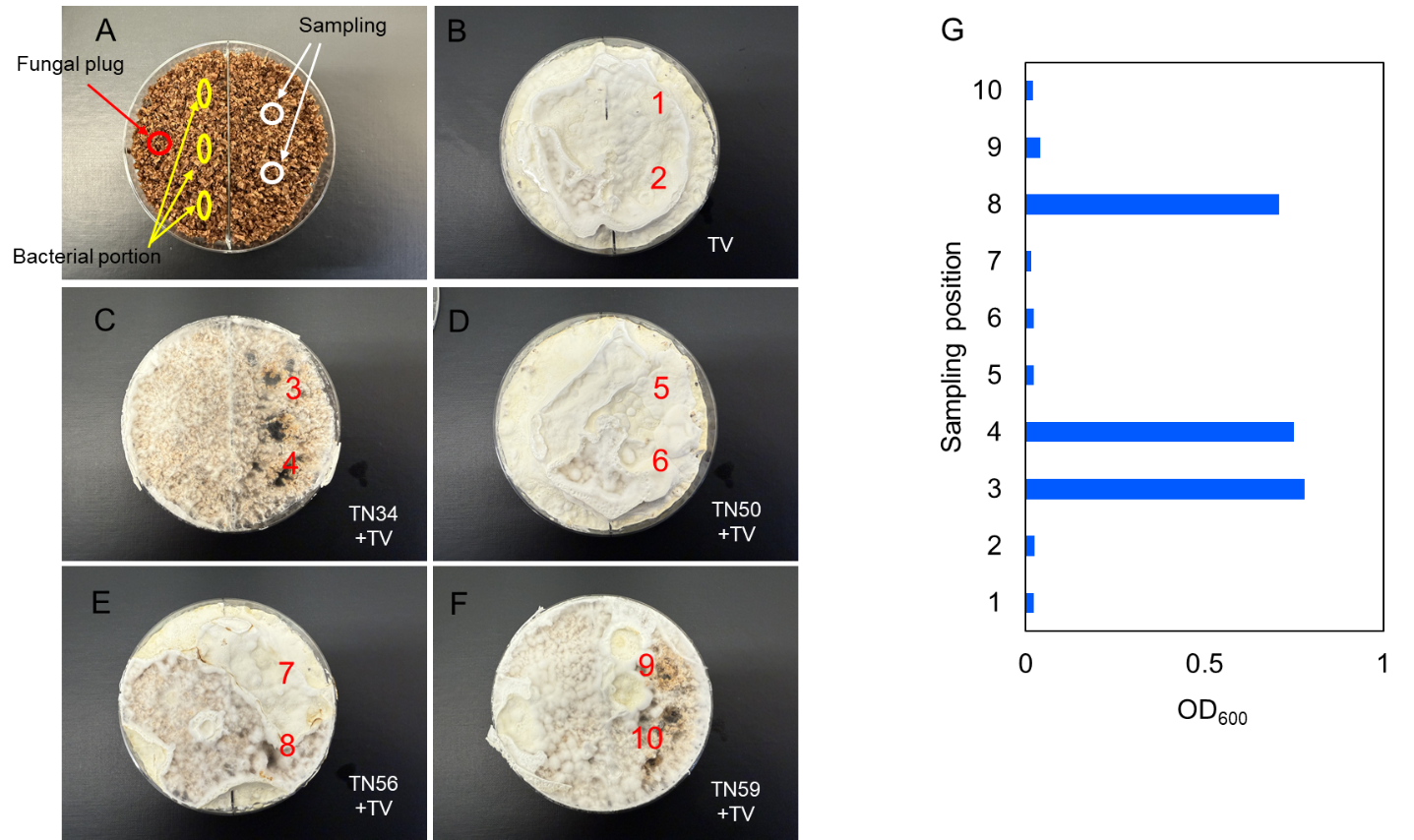


**Fig. S4**

Bacterial migration and survival along fungal hyphae using a dual-chamber plate assay for 90 d. (B-F) Mycelium growth at the time of sampling (90 d after bacterial inoculation) on wood chip medium. (G) OD_600_ in the R2A liquid medium incubated for 3 d to confirm viable bacterial cells of each sampling site. The number indicate the sampling position showing at B-F.


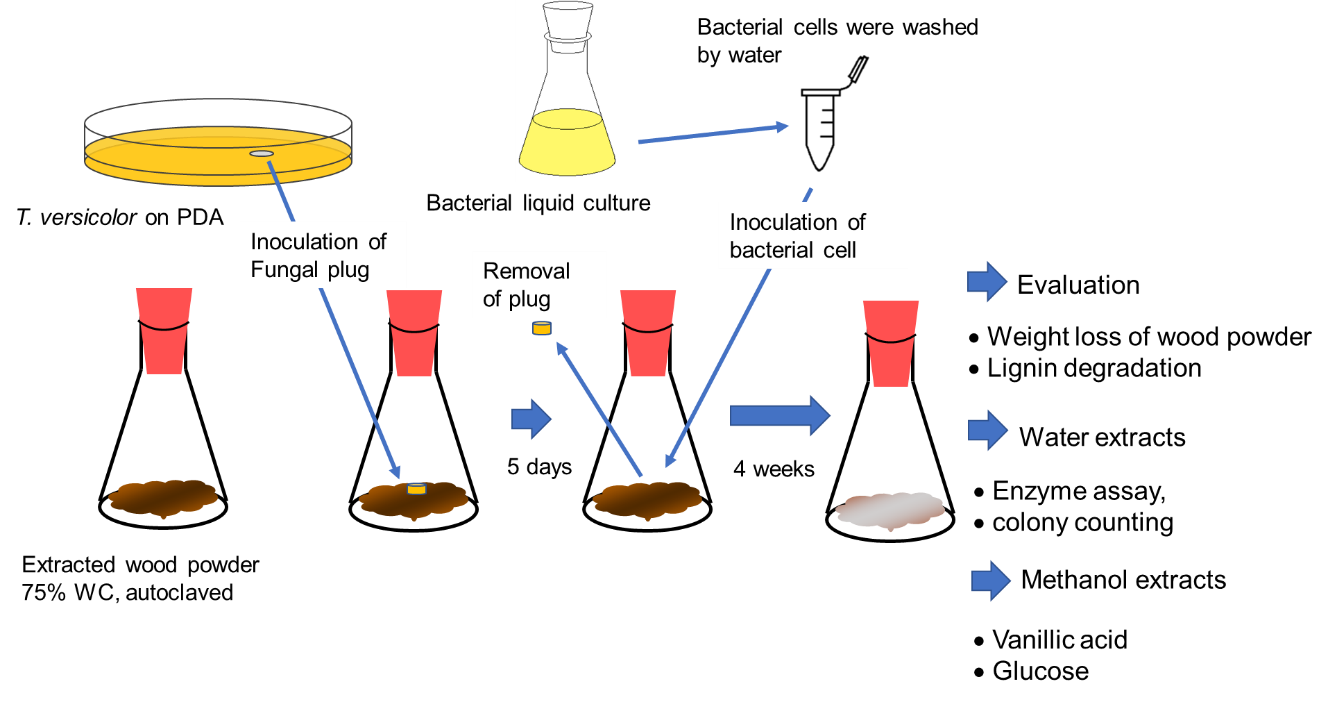


**Fig. S5**

Experimental setup for wood powder co-culture assays *T. versicolor* was pre-inoculated onto sterilized wood powder medium for 5 d prior to bacterial addition. The cultures were incubated for 30 d before sampling. The measured parameters included wood weight loss, lignin degradation, laccase activity, and concentrations of vanillic acid and glucose.


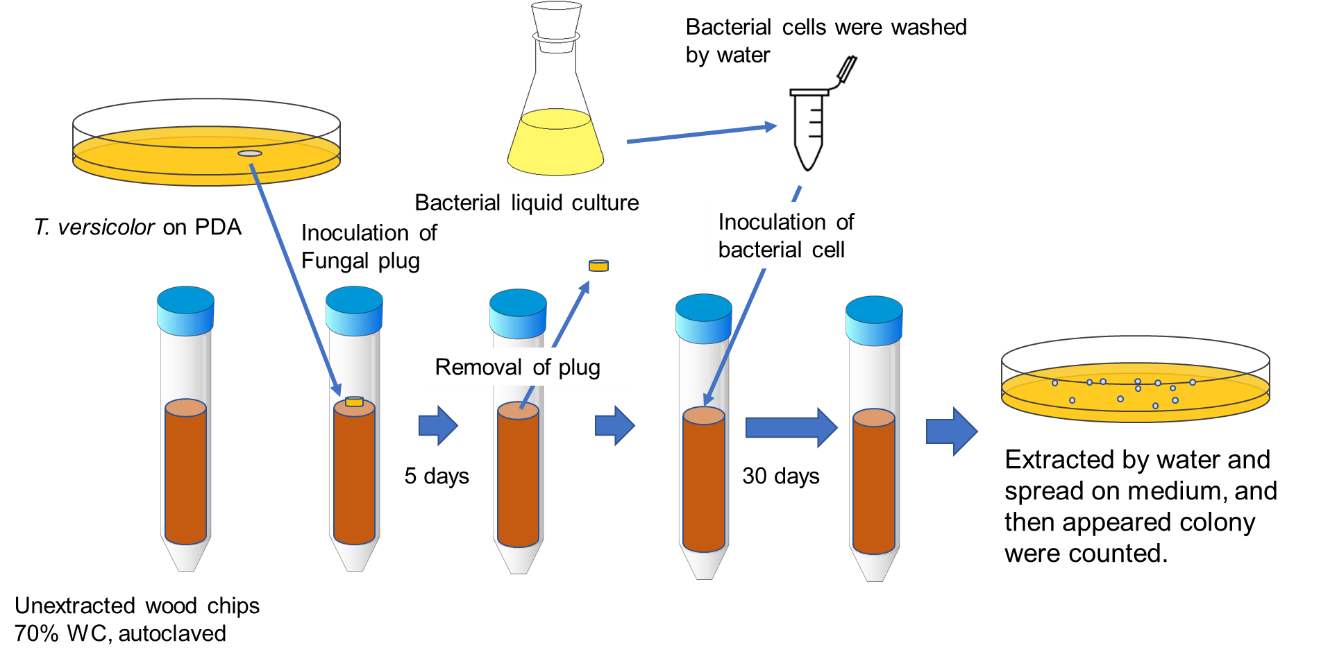


**Fig. S6**

Assessment of bacterial survival using conical tube.
